# The H Index Myth: A Form of Fanaticism or a Simple Misconception?

**DOI:** 10.3390/tomography8030102

**Published:** 2022-05-01

**Authors:** Emilio Quaia, Federica Vernuccio

**Affiliations:** Department of Radiology, University of Padova, Via Giustiniani 2, 35128 Padova, Italy; federica.vernuccio@aopd.veneto.it

Bibliometry represents a branch of library and information sciences that uses statistical methods to analyse publications. Bibliometric indices represent the most relevant component of an individual academic career and are generally used to measure the scientific level of a researcher, considering the average impact of those scientific journals referring to his professional field.

Provided that this may be considered acceptable, can we be sure that the true scientific value of each individual can be measured by bibliometric indices? Are bibliometric indices really related to the actual scientific level, to the level of international collaborations, grant attraction, and educational level of a researcher? Generally, can we use bibliometric indices to classify people working in science as a true biomarker of their scientific value?

There are a plenty of bibliometric indices, including quantitative indices (e.g., number of publications), journal impact indices (e.g., Impact Factor, normalized for 2 or 5 years, and Eigenfactor) and individual impact indices (e.g., h-index and Crown index). The h-index, beside impact factor, is the most famous of these indices and represents an essential reference for many researchers in the academic world. It is particularly promoted and used in the biomedical sciences, a field where the huge number of publications makes any serious qualitative assessment of single researchers almost impossible. Veneration of the h-index achieves a true level of fanaticism since this indicator of quality has become a sort of business card to promote themselves or smile at the lower h-indexes of their colleagues and rivals.

The h-index was invented in 2005 by Jorge Eduardo Hirsch, an Argentine–American professor of physics at the University of California, San Diego, and is defined as the number of articles, N, by an author that have each received at least N citations. Many of us can smile considering that h-index was invented with the declared intention of its creator to show that the number of published papers is not related to the researcher’s impact.

Although the h-index is related to the quality of a researcher (impact factor or citations) and their number of published articles, it is also hampered by many confounding factors [1,2]. The h-index is insensitive to publications that are rarely cited, such as meeting abstracts, and to publications that are frequently cited, such as reviews. If a person has 10 articles that are each cited 100 times, his/her h-index is 10—just like a person who also has 10 articles, but each cited only 10 times. This is because the maximum value of h-index that a scientist can achieve is that of his/her total number of published documents, and because the highly cited papers of a scientist are not properly considered in its calculation. Although highly cited papers are important for the determination of the h-index, once a paper is assigned to the “h-core” category, the number of citations it receives is no longer relevant. This represents a well-known limitation of the h-index but there are several, less mentioned but not less important, limitations which can lead the authors to improper scientific behaviour due to a misconception of the h-index and even to malpractice in science.

The first, less mentioned limitation of the h-index is that the number of authors of an article and the contribution each author makes to the manuscript does not influence the h-index. The h-index does not consider the position of the author within the author list. Considering that the first or second positions in the author list are usually covered by those individuals who are actually the lead authors and the major contributors to the published research, the flattening of authors’ relevance related to the h-index may potentially promote honorary authorship whereby those individuals who want to inflate their h-index ask to be named as authors merely because they hold senior positions within the service or facility where the research occurred and may have helped secure funding.

A second, less known limitation is that the h-index is influenced by self-citation. Self-citation occurs in an article when an author references another of their own publications. This can be a legitimate way to reference earlier findings; but self-citations can sometimes be unduly made in attempt to inflate an individual’s citation count and consequently the h-index. As a consequence, the possibility of inflating the h-index with self-citations lays itself open to fraud and irregularities in publication practice, including an exponential increase in predatory publishers that allow systematic for-profit publication.

A third strong limitation is that the h-index is strongly influenced by the researcher’s scientific age. Consequently, young researchers cannot be compared with older researchers simply by comparing their h-index, since they are penalized by their lower number of published papers and by the lower number of citations due to their shorter scientific life. This scenario may be worse for female researchers as the h-index does not take into account months or years off for parental leave, and a 1-year difference in publication onset or 1-year break in academic publication may result in a “biased” lower academic productivity.

Other important limitations of the h-index include its size-dependent nature, as well as the fact that researchers with selective publication strategies—those who do not publish a very high number of documents but who do attain a high impact due to few studies with huge scientific impact—can be unfairly assessed through the h-index. A scientist or a researcher who, for whatever reason, has had very few but very influential articles, receives a low h-index and vice versa. With the need to increase their h-index to boost an author’s academic reputation, authors may choose to deviate their research towards research topics that will likely receive a higher number of citations, rather than choosing more clinically relevant research that will likely result in a lower number of citations. As an example, in the last 5 years the number of papers published in PubMed on “radiomics”, “artificial intelligence" or "texture analysis” has tripled; thus, if an author decides to publish on this topic, he knows that the likelihood of being cited will be higher as compared with other research topics for which the trend is approximately stable over time. This may unduly favour a biased research approach, primarily considering topics that are popular and that will likely receive many citations, and to rapidly publish as many papers as possible even with less data, thus resulting in meaningless original research studies.

Finally, the financial impact of the h-index must be acknowledged. The h-index cannot take into account the specific field of a researcher. Researchers working in nonmainstream areas will have lower h-values as compared to those working in highly topical areas. However, in the context of obtaining grant funding, researchers of different medical fields may compete for the same funding with similar projects, but the competition may unduly penalize the researcher working in nonmainstream areas, unless bibliometric indicators to account for interdisciplinary differences are used. This may result in a biased allocation of funding towards researchers working in highly topical areas.

Considering all these issues, is there a solution? Presently, there are no perfect bibliometric indices to describe the scientific impact of a researcher. The h-index is certainly abused as a score of the scientific value of a researcher and presents several intrinsic limitations which should be acknowledged before promoting people to higher academic ranks and career stages.

## Data Availability

Not applicable.

## References

[1] Costas R., Bordons M. (2007). The h-index: Advantages, limitations and its relation with other bibliometric indicators at the micro level. J. Informetr..

[2] Ding J., Liu C., Kandonga G.A. (2020). Exploring the limitations of the h-index and h-type indexes in measuring the research performance of authors. Scientometrics.

